# Association of the GLM7 composite metabolic index with gestational diabetes mellitus in early pregnancy: a secondary analysis of a Korean prospective pregnancy cohort

**DOI:** 10.3389/fnut.2026.1907985

**Published:** 2026-09-15

**Authors:** Qinfei Zhao, Weiquan Hu, Xuxiang Xi, Lingyu Wu, Suping Hu, Yi Liu, Yingjie Chen

**Affiliations:** 1 Department of Laboratory Medicine, The First Affiliated Hospital of Gannan Medical University, Ganzhou, Jiangxi, China; 2 Department of Joint Surgery, Ganzhou People's Hospital, Ganzhou, Jiangxi, China; 3 The First School of Clinical Medicine, Gannan Medical University, Ganzhou, Jiangxi, China; 4 Department of Emergency, The First Affiliated Hospital of Gannan Medical University, Ganzhou, Jiangxi, China; 5 Medicinal Plant Research and Development Center of Hubei Province, Hubei University of Traditional Chinese Medicine, Wuhan, Hubei, China; 6 Department of Emergency, Shishi Municipal Hospital, Quanzhou, Fujian, China

**Keywords:** cohort study, early pregnancy, gestational diabetes mellitus, GLM7, insulin resistance

## Abstract

**Background:**

Surrogate indices such as the triglyceride-glucose (TyG) index are widely used to estimate insulin resistance (IR), yet their ability to discriminate incident gestational diabetes mellitus (GDM) in early gestation remains suboptimal. This study evaluated whether the previously developed composite metabolic index GLM7 was correlated with subsequent GDM in Korean singleton pregnant women.

**Methods:**

In this study, we analyzed 582 singleton pregnancies from a Korean pregnancy registry. Multivariable logistic regression and restricted cubic splines (RCS) were used to evaluate the association between GLM7 and GDM. Discriminative performance was assessed by receiver operating characteristic (ROC) curve analysis, and pairwise comparisons were performed using DeLong's test. Robustness was assessed through sensitivity analyses, subgroup analyses, and E-value calculations.

**Results:**

In the fully adjusted model, GLM7 was independently associated with incident GDM (OR 14.27, 95% CI 5.01–40.67; *p* < 0.001). RCS analysis showed no evidence of departure from linearity (*p* for non-linearity = 0.266). The association persisted across sensitivity and subgroup analyses. The E-value of 28.03 suggested that only a very strong unmeasured confounder could nullify the observed association. ROC analysis revealed an AUC of 0.839 (95% CI 0.767–0.912) for GLM7. Pairwise DeLong comparisons showed significantly higher AUCs for GLM7 than for HOMA-IR and for first-trimester fasting plasma glucose (FPG), while differences compared with the TyG index, fatty liver index (FLI), and hepatic steatosis index (HSI) were not statistically significant. Within this cohort, the optimal GLM7 threshold was 6.932, yielding 77.8% sensitivity and 78.2% specificity.

**Conclusions:**

GLM7 was independently associated with subsequent GDM.

## 1 Introduction

Gestational diabetes mellitus (GDM), defined as hyperglycaemia first recognized during pregnancy, is the most common metabolic disorder of pregnancy, affecting approximately 15% of pregnancies worldwide and imposing substantial health burdens on both mothers and their offspring (1–3). Its etiology is multifactorial and involves interactions among genetic, epigenetic, and environmental factors (4). Established risk factors include maternal obesity, advanced maternal age, multiparity, and excessive gestational weight gain (5, 6). GDM substantially increases the risk of adverse perinatal outcomes, including pre-eclampsia, macrosomia, and cesarean delivery, while also increasing the long-term risk of metabolic disorders, such as obesity, cardiovascular disease (CVD), and type 2 diabetes mellitus (T2DM), in both mothers and their offspring (7, 8). Accumulating evidence suggests that early identification and intervention for GDM risk in early gestation can reduce adverse perinatal and postnatal outcomes and improve long-term metabolic health (9, 10). However, individual biomarkers measured during early pregnancy, such as fasting plasma glucose (FPG) or isolated lipid parameters, show limited sensitivity and specificity, restricting accurate risk stratification, and highlighting the necessity for more comprehensive and cost-effective screening methods (11).

Metabolic dysfunction during early pregnancy is consistently associated with the subsequent development of GDM (11). Women who later develop GDM frequently exhibit characteristic metabolic alterations during the first-trimester, including elevated liver enzymes [e.g., alanine aminotransferase (ALT) and gamma-glutamyl transferase (GGT)], dyslipidaemia (e.g., elevated triglycerides [TG] and free fatty acids [FFAs]), and altered adipokine profiles, particularly reduced adiponectin (ADPN) (11–14). However, the reported associations between individual liver enzymes, lipid profiles, and GDM risk have been inconsistent across different populations, likely reflecting the complex and interrelated nature of metabolic dysfunction. Notably, nonalcoholic fatty liver disease (NAFLD), a hepatic manifestation of systemic metabolic dysfunction, is closely associated with insulin resistance (IR) and an increased risk of GDM (15, 16). However, routine imaging for NAFLD is of limited feasibility during pregnancy, and reliance on single biomarkers inadequately captures the multidimensional metabolic derangements preceding GDM. Consequently, composite metabolic indices that integrate multiple metabolic pathways may provide a more comprehensive assessment of early metabolic dysfunction and improve early GDM risk stratification.

In recent years, composite metabolic indices have gained increasing attention as practical tools for integrating multiple biomarkers into a single, clinically interpretable metric. For example, the ZJU index, a composite index originally developed to identify NAFLD, incorporates FPG, TG, the ALT/aspartate aminotransferase (AST) ratio, and body mass index (BMI) to reflect overall metabolic status (17–20). Compared with individual markers, composite metabolic indices have demonstrated improved performance in identifying metabolic dysfunction, including NAFLD and related metabolic disorders (18–23). However, their application during pregnancy remains insufficiently investigated. In particular, the ZJU index does not incorporate adipokines or FFAs, both of which are important mediators of gestational metabolic adaptation. Considering that both GDM and T2DM originate from similar underlying processes involving IR and glucose-lipid metabolism disturbances, and that lipid and adipokine imbalances often precede hyperglycemia in early pregnancy (24), further evaluation of composite metabolic indices that integrate hepatic, lipid and adipokine-related metabolic information may improve early GDM risk stratification.

Recently, Wang et al. (25) developed and validated the glycolipid metabolism 7-factor (GLM7) index, a composite metabolic index integrating age, TG, pre-pregnancy BMI, FPG, insulin, low-density lipoprotein cholesterol (LDL), and high-density lipoprotein cholesterol (HDL), in 26,289 participants from the National Health and Nutrition Examination Survey (NHANES). The index was subsequently externally validated in the China Health and Retirement Longitudinal Study (CHARLS) cohort and demonstrated favorable performance for identifying cardiometabolic multimorbidity. However, its applicability during early pregnancy, a period characterized by profound metabolic adaptation, remains unknown. To our knowledge, no previous study has evaluated the performance of GLM7 in pregnant women or investigated its association with incident GDM. Therefore, this study provides the first independent pregnancy-specific evaluation of the previously published GLM7 index and extends its application to a clinically distinct outcome. Because GLM7 integrates demographic, glycemic, insulinemic, and lipid-related variables, it may capture broader metabolic disturbances preceding GDM than individual biomarkers alone. We hypothesized that higher GLM7 values at 10–14 weeks of gestation would be associated with an increased risk of subsequent GDM. Accordingly, this study aimed to evaluate the association between GLM7 and incident GDM, characterize its dose-response relationship, and assess its discriminative performance relative to established metabolic markers and composite metabolic indices, including the triglyceride-glucose (TyG) index, fatty liver index (FLI), hepatic steatosis index (HSI), homeostatic model assessment of insulin resistance (HOMA-IR), and FPG.

## 2 Methods

### 2.1 Study design

This prospective cohort study analyzed data from participants enrolled in the “Fatty Liver in Pregnancy” registry (ClinicalTrials.gov NCT02276144; registered 28 October 2014), conducted at Seoul National University Boramae Medical Center and Incheon Seoul Women's Hospital (26).

### 2.2 Data source

The data for this secondary analysis were obtained from Lee et al.'s (26) PLoS ONE article, “Nonalcoholic fatty liver disease is a risk factor for large-for-gestational-age birthweight” (https://journals.plos.org/plosone). The dataset is distributed under the Creative Commons Attribution License (CC BY), which permits unrestricted reuse, distribution and reproduction in any medium, provided that the original authors and source are appropriately credited. We thank the original investigators and participants for making these data publicly available.

### 2.3 Study participants

The parent study was approved by the Institutional Review Board of Seoul National University Boramae Medical Center and the Public Institutional Review Board of the Korean Ministry of Health and Welfare. Therefore, no additional ethical approval was required for this secondary analysis. Written informed consent was obtained from all participants at enrollment, and the original study was conducted in accordance with the Declaration of Helsinki. Reporting followed the STROBE checklist (27).

Originally, between November 2014 and July 2016, 663 singleton pregnancies that met the initial eligibility criteria were screened. All individuals underwent liver ultrasound examinations at 10–14 weeks of pregnancy (26). Forty women were subsequently excluded because of loss to follow-up or delivery before 34 weeks, leaving 623 participants for initial consideration. After applying data-completeness criteria, we excluded 13 participants with missing GDM status, 23 without HSI values, 1 with missing FPG, 3 lacking FLI information, and 1 without FFA measurements. The final analytic cohort comprised 582 singleton pregnancies, of which 36 developed GDM and 546 did not. The primary analyses were performed using a complete-case dataset to ensure complete information on the exposure (GLM7), outcome (GDM), and all prespecified covariates. Multiple imputation was not performed because the proportion of missing data after cohort eligibility screening was low, and the primary outcome (GDM status) could not be imputed reliably when it was missing. Details of participant selection and exclusions are presented in Figure 1.

**Figure 1:**
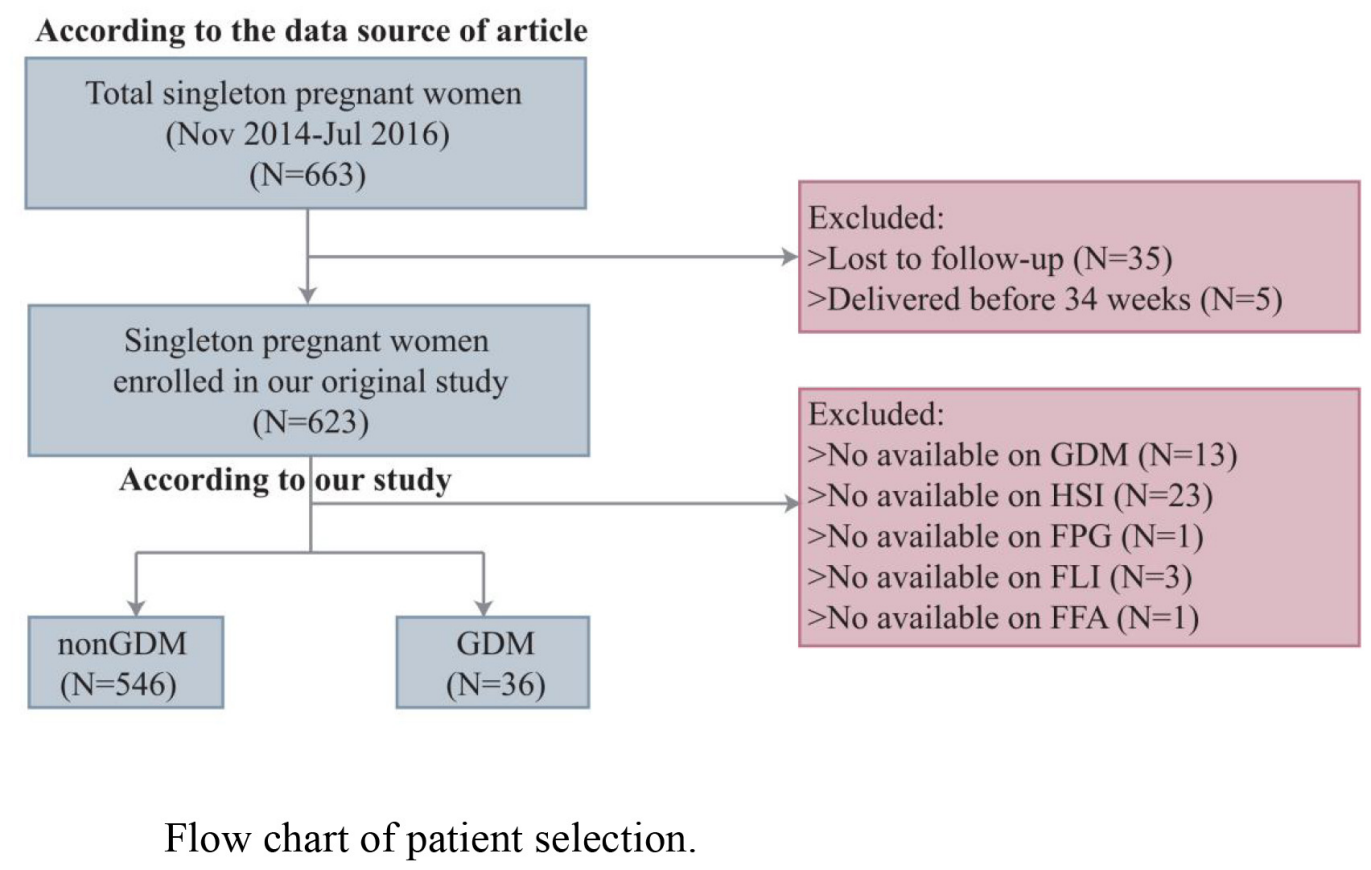
Flow chart of patient selection.

### 2.4 Data collection

Trained clinical personnel collected baseline demographic and clinical characteristics using a standardized questionnaire, including maternal age, parity, alcohol use, pre-pregnancy BMI, and history of diabetes. After an overnight-fast (>8 h), venous blood samples were collected at 10–14 weeks' gestation for biochemical assays. Laboratory measurements included ALT, AST, total cholesterol (TC), gamma-glutamyl transferase (GGT), TG, HDL, LDL, FPG, and insulin. Hepatic steatosis (HS) was assessed by ultrasonography and graded semi-quantitatively on a scale from 0 to 3 according to established criteria (28). For the sensitivity analyses, NAFLD was defined as a binary variable, with an HS grade ≥1 indicating the presence of NAFLD and grade 0 indicating its absence. IR was estimated using HOMA-IR (29). GDM was assessed at 24–28 weeks of gestation via a 75 g oral glucose tolerance test (OGTT) and diagnosed according to the International Association of Diabetes and Pregnancy Study Groups (IADPSG) criteria: FPG ≥ 5.1 mmol/L, 1-h post-load glucose ≥ 10.0 mmol/L, or 2-h post-load glucose ≥ 8.5 mmol/L (10, 30). Additional methodological details and variable definitions are provided in the original publication (26).

### 2.5 Assessment of the GLM7 index

The GLM7 index was calculated according to the previously published formula using seven first-trimester maternal parameters: GLM7 = log_10_ [Age (years) × BMI (kg/m^2^) × FPG (mg/dL) × Insulin (pmol/L) × TG (mmol/L) × LDL (mmol/L) / HDL (mmol/L)] (25). Fasting insulin values were converted from μIU/mL to pmol/L (1 μIU/mL = 6.0 pmol/L) (31). Lipid parameters were converted from mg/dL to mmol/L using standard factors: TG (÷ 88.57) and cholesterol (÷ 38.67).

For comparison analyses, several established metabolic indices were also evaluated. The TyG index was calculated according to the original published formula: TyG = ln [TG (mg/dL) × FPG (mg/dL)/2] (32). HOMA-IR, the FLI, and the HSI were available in the original cohort dataset and had been calculated by the original investigators according to their respective published formulas (26), which are presented below for completeness. HOMA-IR = fasting insulin (μIU/mL) × FPG (mmol/L) / 22.5 (33). FLI was calculated according to Bedogni et al. (34): FLI = [exp (0.953 × ln (TG) + 0.139 × BMI + 0.718 × ln (GGT) + 0.053 × waist circumference (WC) – 15.745) / (1 + exp (0.953 × ln (TG) + 0.139 × BMI + 0.718 × ln (GGT) + 0.053 × WC – 15.745))] × 100, where TG is measured in mg/dL, BMI in kg/m^2^, GGT in U/L, and WC in cm. The HSI was calculated according to Lee et al. (35): HSI = 8 × [ALT (IU/L) / AST (IU/L)] + BMI (kg/m^2^) + 2.

All biochemical measurements were performed on venous samples obtained after an overnight-fast (> 8 h) at 10–14 weeks' gestation. Samples were centrifuged and stored at −70 °C until analysis. The outcome variable was GDM.

### 2.6 Covariates

Covariate selection was guided by directed acyclic graphs (DAGs) (Supplementary Figure S1) and prior literature, with the number of adjusted variables constrained by the “10 events per variable” principle (36 GDM events permitted ≤ 3–4 covariates) (26, 36–38). The final multivariable model adjusted for parity, TC, ADPN, and FFA. The assessment of multicollinearity among independent variables was conducted using the variance inflation factor (VIF) (39). Additional details regarding the original data collection procedures are described in the parent publication (26).

### 2.7 Statistical analysis

GDM status was used to summarize baseline characteristics. Participants were ranked by GLM7 and divided into three approximately equal-sized tertiles denoted as low (T1), intermediate (T2), and high (T3) exposure (25, 40–42). Continuous variables with an approximately normal distribution were presented as the mean ± standard deviation (SD), whereas skewed variables were summarized as the median (interquartile range). Categorical variables were expressed as n (%). Group comparisons for normally distributed continuous variables were conducted using one-way ANOVA, whereas non-normally distributed continuous data were analyzed with the Kruskal-Wallis test. Categorical outcomes were assessed using Pearson's chi-square test. For the tertile analyses, a *p* for trend was calculated by assigning ordinal scores (1, 2, and 3) to increasing GLM7 tertiles and entering the resulting variable as a continuous term in the multivariable logistic regression model. The statistical significance of the linear trend was assessed using the Wald test for the regression coefficient of the ordinal variable (43).

The associations of GLM7 with GDM were assessed using multivariable logistic regression and restricted cubic splines (RCS). Multivariable models were fitted sequentially as follows: crude (unadjusted); Model I (adjusted for parity); Model II (parity + FFA); Model III (parity + FFA + TC); and Model IV (parity + FFA + TC + ADPN). Odds ratios (ORs) with 95% confidence intervals (CIs) were reported. Fully adjusted RCS models were used to characterize the dose-response relationship and assess potential non-linearity. Subgroup analyses were performed after stratification by age, HOMA-IR, HS, and pre-pregnancy BMI (44). Continuous stratification variables were categorized using clinically relevant cut-points, and within each stratum, models were adjusted for the same covariates except the stratification factor (6). Interaction was tested by comparing models with and without multiplicative interaction terms using likelihood-ratio tests.

We performed multiple sensitivity analyses to assess robustness. First, GLM7 was analyzed categorically (tertiles). To evaluate the robustness of the primary findings with respect to obesity and underlying HS (26, 45), sensitivity analyses were performed by excluding participants with pre-pregnancy BMI ≥ 25 kg/m^2^ and, separately, participants with sonographic HS grade ≥ 1. We also evaluated models with NAFLD included as an additional covariate. E-values were determined to measure the least degree of association an unmeasured confounder would require with both the exposure and the outcome to nullify the observed associations (46).

Discriminative performance was evaluated using receiver operating characteristic (ROC) analysis by estimating the area under the curve (AUC) and identifying the optimal operating point using Youden's index for GLM7 and the comparator metabolic markers. Pairwise comparisons between GLM7 and each comparator marker were performed using DeLong's test. To explore whether GLM7 provided additional discriminatory information beyond established composite metabolic indices, covariate-adjusted ROC analyses were additionally conducted as sensitivity analyses using predicted probabilities derived from separate multivariable logistic regression models with an identical covariate structure comprising parity, TC, FFA, and ADPN. Each metabolic marker or composite index was added individually to the same base model. The sample size was determined by the available dataset; therefore, *a priori* power calculation was not performed.

Statistical analyses were performed using R software (version 4.2.2; R Foundation for Statistical Computing, Vienna, Austria) and Free Statistics software (version 2.5; Beijing, China; available at http://www.clinicalscientists.cn/freestatistics), a graphical statistical analysis platform that provides a user-friendly interface for regression analyses, RCS models, and other commonly used epidemiological statistical procedures. All tests were two-sided and significance was defined as *p* < 0.05.

## 3 Results

### 3.1 Clinical characteristics of study participants

Table 1 displays the demographic and clinical features stratified by GDM status. Out of 582 participants, 36 (6.2%) were diagnosed with GDM. The mean age of mothers was 32.1 years. Women who developed GDM showed a less favorable metabolic profile during early pregnancy than those who did not, including higher FPG, fasting insulin, FFA, HOMA-IR, ALT, GGT, and TG levels, lower adiponectin and HDL concentrations, and a higher prevalence of HS on ultrasonography (all *p* < 0.05). Baseline characteristics of the included and excluded participants are presented in Supplementary Table S1. Overall, the two groups were broadly comparable with respect to most baseline characteristics. However, excluded participants had modestly higher first-trimester FPG and FFA levels than those included in the final analysis.

**Table 1:** Clinical characteristics of study participants.

Characteristic	Total (*N* = 582)	Without GDM (*N* = 546)	With GDM (*N* = 36)	*p*-value
Age (years)	32.1 ± 3.8	32.0 ± 3.8	32.6 ± 3.6	0.431
BMI (kg/m^2^)	22.0 ± 3.5	21.8 ± 3.2	25.8 ± 5.2	< 0.001
AST (IU/L)	17.8 ± 8.1	17.7 ± 8.2	19.7 ± 7.3	0.154
ALT (IU/L)	11.0 (8.0, 15.0)	11.0 (8.0, 14.0)	15.0 (10.0, 25.5)	0.003
GGT (IU/L)	14.0 ± 8.5	13.8 ± 8.4	17.7 ± 8.9	0.007
TC (mg/dL)	172.9 ± 27.1	172.5 ± 26.9	180.2 ± 29.7	0.096
TG (mg/dL)	119.0 ± 47.5	115.3 ± 41.7	176.1 ± 83.1	< 0.001
HDL (mg/dL)	64.9 ± 13.5	65.3 ± 13.3	58.9 ± 16.5	0.006
LDL (mg/dL)	84.1 ± 21.7	84.1 ± 21.1	84.0 ± 28.7	0.981
FPG (mg/dL)	76.9 ± 9.7	76.4 ± 9.0	84.6 ± 15.2	< 0.001
Insulin (μIU/mL)	8.4 (5.4, 11.6)	8.1 (5.3, 11.2)	15.3 (8.9, 21.9)	< 0.001
FFA (μEq/L)	648.3 ± 272.3	642.0 ± 269.1	743.6 ± 304.9	0.03
GLM7	6.6 ± 0.5	6.6 ± 0.4	7.2 ± 0.4	< 0.001
parity				0.963
No	305 (52.4)	286 (52.4)	19 (52.8)	
Yes	277 (47.6)	260 (47.6)	17 (47.2)	
NAFLD				< 0.001
No	472 (81.1)	456 (83.5)	16 (44.4)	
Yes	110 (18.9)	90 (16.5)	20 (55.6)	
ADPN (ng/mL)	5,026.2 (2,821.3, 8,051.0)	5,359.8 (3,129.0, 8,238.2)	1,943.3 (1,297.8, 2,864.2)	< 0.001
HOMA-IR	1.5 (1.0, 2.3)	1.5 (0.9, 2.2)	2.9 (1.7, 4.7)	< 0.001
Hepatic steatosis				< 0.001
Grade 0	472 (81.1)	456 (83.5)	16 (44.4)	
Grade 1	85 (14.6)	76 (13.9)	9 (25)	
Grade 2	17 (2.9)	9 (1.6)	8 (22.2)	
Grade 3	8 (1.4)	5 (0.9)	3 (8.3)	
FLI	11.5 (6.1, 22.7)	10.9 (6.0, 20.1)	39.8 (24.5, 68.7)	< 0.001
HSI	30.3 ± 4.7	30.0 ± 4.4	35.7 ± 5.8	< 0.001
TyG	8.4 ± 0.4	8.3 ± 0.4	8.8 ± 0.4	< 0.001

BMI, body mass index; TG, triglyceride; TC, total cholesterol; HDL, high-density lipoprotein cholesterol; LDL, low-density lipoprotein cholesterol; FPG, fasting plasma glucose; ALT, alanine aminotransferase; GGT, gamma-glutamyl transferase; AST, aspartate aminotransferase; FFA, free fatty acid; NAFLD, nonalcoholic fatty liver disease; ADPN, adiponectin; HOMA-IR, homeostatic model assessment of insulin resistance; FLI, fatty liver index; HSI, hepatic steatosis index; TyG, triglyceride-glucose index; GDM, gestational diabetes mellitus. Values are reported as mean ± SD, median (Q1–Q3), or N (%). NAFLD was defined as hepatic steatosis grade ≥ 1 on ultrasonography.

### 3.2 Relationship between GLM7 index and GDM

All variables exhibited VIFs < 1.2, indicating no multicollinearity (Supplementary Table S2). The Box-Tidwell test confirmed a linear relationship between GLM7 and the risk of GDM (*p* = 0.326), meeting the linearity requirement for logistic regression (Supplementary Table S3).

GLM7 was evaluated as a continuous variable and also grouped into tertiles. As shown in Table 2, higher GLM7 was strongly associated with incident GDM. As a continuous predictor, GLM7 yielded a crude OR of 24.96 (95% CI 9.66–64.45; *p* < 0.001) and maintained its significance even after sequential adjustments for parity, FFA, TC and ADPN (Model IV OR 14.27, 95% CI 5.01–40.67; *p* < 0.001). According to tertile analyses, participants in the highest GLM7 tertile (T3) had substantially greater odds of developing GDM than those in the lowest tertile (T1) (crude OR 17.56, 95% CI 4.13–74.60; *p* < 0.001; Model IV OR 8.14, 95% CI 1.80–36.77; *p* = 0.006), whereas the intermediate tertile (T2) did not show a significant elevation. The models demonstrated a clear dose-response trend (*p* for trend < 0.001). The corresponding E-value (28.03) indicates that an unmeasured confounder would need to be very strongly associated with both GLM7 and GDM to fully explain the observed association. RCS analysis supported a linear dose-response between GLM7 and GDM risk (*p* for non-linearity = 0.266; Figure 2).

**Table 2:** Relationship between GLM7 and GDM risk in different models.

Exposure	Crude model (OR, 95% CI, *p*)	Model I (OR, 95% CI, *p*)	Model II (OR, 95% CI, *p*)	Model III (OR, 95% CI, *p*)	Model IV (OR, 95% CI, *p*)	E-value
GLM7	24.96 (9.66–64.45) < 0.001	26.04 (9.99–67.88) < 0.001	24.68 (9.43–64.60) < 0.001	27.39 (10.14–74.03) < 0.001	14.27 (5.01–40.67) < 0.001	28.03
GLM7 tertile						
T1	1.0	1.0	1.0	1.0	1.0	
T2	2.02 (0.37–11.17) 0.420	2.04 (0.37–11.26) 0.414	2.09 (0.38–11.58) 0.398	2.13 (0.38–11.87) 0.389	1.55 (0.27–8.82) 0.621	2.47
T3	17.56 (4.13–74.60) < 0.001	18.04 (4.23–76.83) < 0.001	17.54 (4.11–74.84) < 0.001	18.08 (4.13–79.15) < 0.001	8.14 (1.80–36.77) 0.006	15.76
*P* for trend	< 0.001	< 0.001	< 0.001	< 0.001	< 0.001	

Crude model: no covariates were adjusted. Model I: adjusted for parity. Model II: adjusted for parity, and FFA. Model III: adjusted for parity, FFA, and TC. Model IV: adjusted for parity, FFA, TC, and ADPN. OR, odds ratio; CI, confidence interval.

**Figure 2:**
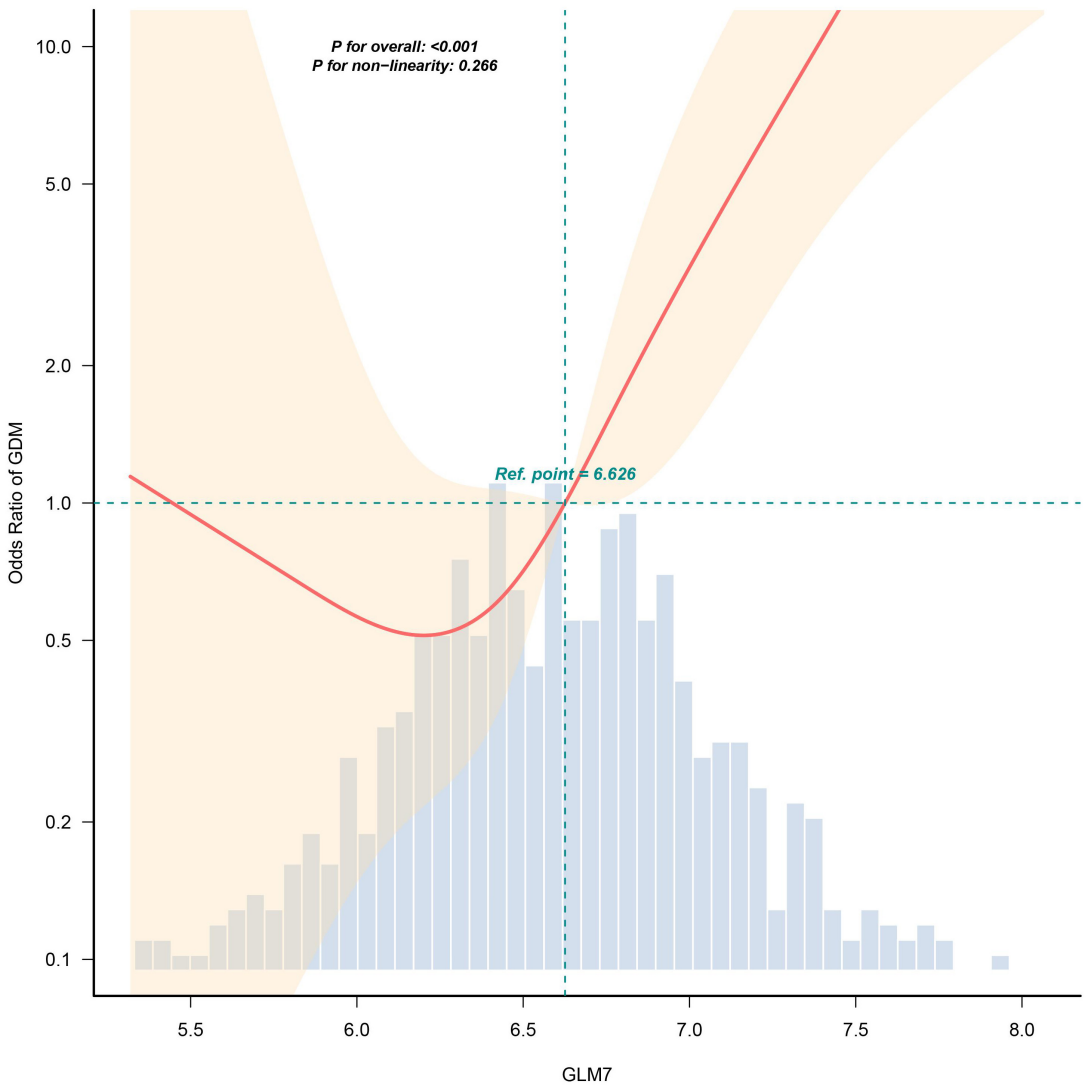
Restricted cubic spline showing the dose-response relationship between GLM7 and GDM. Adjusted for parity, ADPN, TC, and FFA.

### 3.3 Sensitivity analyses

When GLM7 was modeled as a continuous variable, its positive relationship with GDM remained consistent across all sensitivity analyses (Model I OR 7.05, 95% CI 1.72–28.90, *p*=0.007; Model II OR 7.31, 95% CI 1.86–28.72, *p*=0.004; Model III OR 10.25, 95% CI 3.39–30.94, *p* < 0.001) (Table 3). In the tertile analyses, participants in the highest GLM7 tertile (T3) had the greatest risk of GDM compared with those in the lowest tertile (T1), reaching statistical significance in Model III (OR 5.61, 95% CI 1.19–26.35; *p* = 0.029), whereas the intermediate tertile (T2) was not significantly associated with GDM. A significant dose-response relationship was observed across the sensitivity models (*p* for trend = 0.046, 0.029 and 0.006 for Models I–III, respectively), supporting the robustness of the observed association between GLM7 and incident GDM.

**Table 3:** Relationship between GLM7 and GDM risk in different sensitivity analyses.

Exposure	Model I (OR, 95% CI, *p*)	Model II (OR, 95% CI, *p*)	Model III (OR, 95% CI, *p*)
GLM7	7.05 (1.72–28.90) 0.007	7.31 (1.86–28.72) 0.004	10.25 (3.39–30.94) < 0.001
GLM7 tertile			
T1	1.0	1.0	1.0
T2	1.15 (0.18–7.22) 0.885	1.21 (0.19–7.57) 0.837	1.47 (0.26–8.42) 0.663
T3	3.94 (0.78–19.97) 0.098	4.66 (0.92–23.70) 0.063	5.61 (1.19–26.35) 0.029
*p* for trend	0.046	0.029	0.006

Model I: Adjusted for parity, FFA, TC, and ADPN in participants with a pre-pregnancy BMI < 25 kg/m^2^ (*N*=486).

Model II: Sensitivity analysis included individuals with grade 0 hepatic steatosis (*N*=472), adjusting for parity, FFA, TC, and ADPN. OR: odds ratio; CI: confidence interval.

Model III: Sensitivity analysis with NAFLD included as a covariate. Adjusted for parity, FFA, TC, NAFLD, and ADPN.

### 3.4 Subgroup analysis

We investigated possible effect modification by conducting stratified analyses based on age, HOMA-IR, pre-pregnancy BMI, and HS. Within each stratum, models were adjusted for the same covariates except the stratification variable. The numbers of participants and GDM events for each subgroup are presented in Figure 3 and Supplementary Table S4. Because the number of GDM events within several subgroups was limited, the corresponding effect estimates should be interpreted cautiously. Nevertheless, the direction and magnitude of the association were generally consistent across all predefined subgroups. No statistically significant interactions were observed for any of the stratification variables (all *p* for interaction > 0.05), indicating no evidence of effect modification.

**Figure 3:**
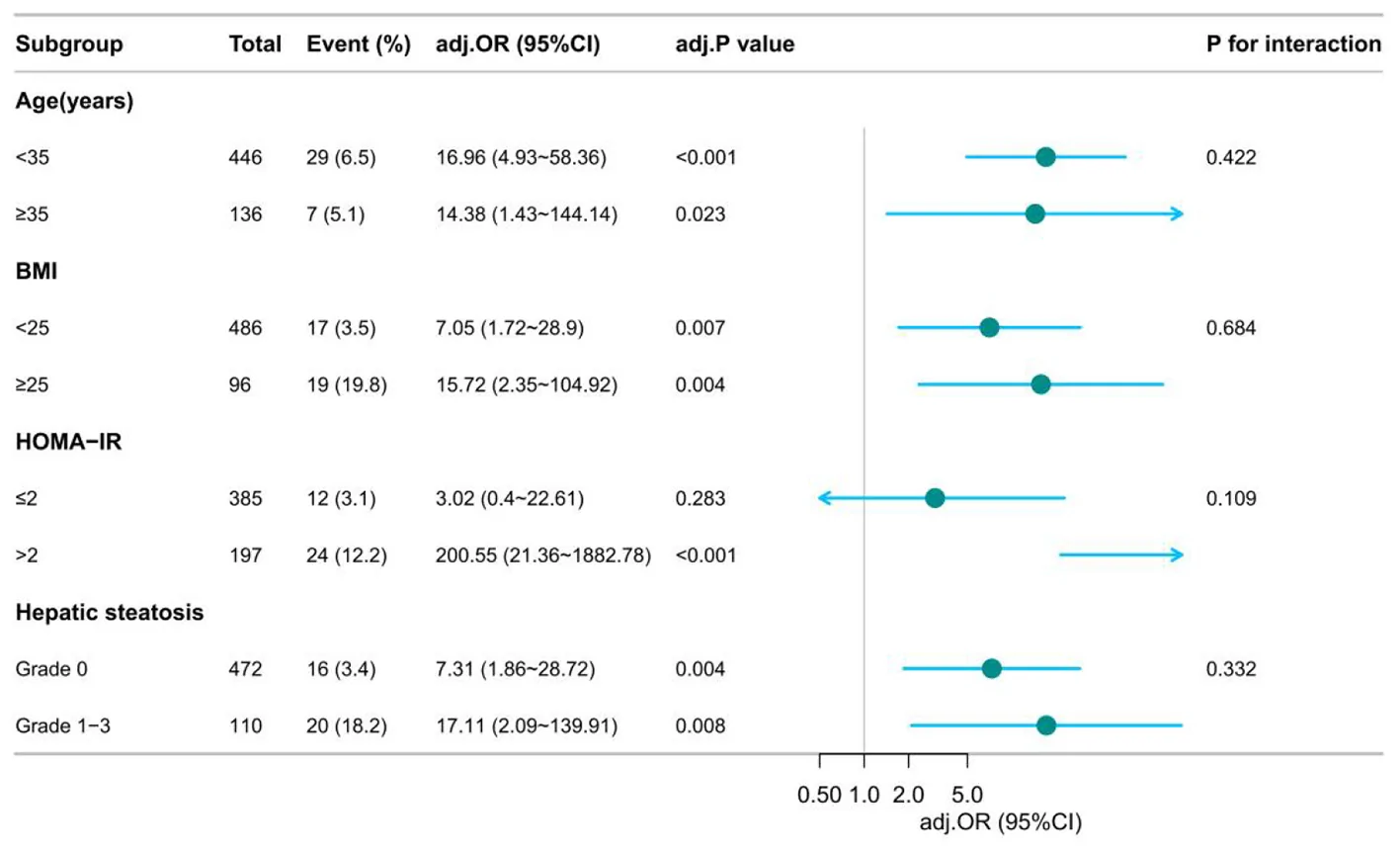
Subgroup analysis of the association between GLM7 and GDM risk.

### 3.5 ROC analysis

The discriminative performance of GLM7 for incident GDM was assessed by ROC analysis. GLM7 achieved an AUC of 0.839 (95% CI 0.767–0.912), indicating good discrimination within this cohort (Table 4 and Figure 4). Among the evaluated metabolic markers, GLM7 showed the highest observed AUC. In the unadjusted ROC analyses, pairwise DeLong comparisons demonstrated significantly higher AUCs for GLM7 than for HOMA-IR and FPG, whereas the differences compared with the TyG index, FLI, and HSI were not statistically significant. After covariate adjustment, GLM7 retained the highest numerical AUC (Supplementary Figure S2); however, none of the pairwise AUC differences reached statistical significance (all *p* > 0.05; Supplementary Table S5). The optimal GLM7 threshold determined by Youden's index was 6.932, corresponding to a sensitivity of 77.80% and a specificity of 78.20%. Because this threshold was derived and evaluated within the same cohort, it should be regarded as exploratory and requires external validation before clinical application.

**Table 4:** AUC for each assessed parameter in determining GDM.

Variables	AUC (95% CI)	Best threshold	specificity	sensitivity	Youden index
GLM7	0.839 (0.767–0.912)	6.932	0.782	0.778	0.560
TyG index	0.807 (0.735–0.880)	8.632	0.788	0.722	0.510
FLI	0.814 (0.728–0.900)	23.328	0.806	0.806	0.612
HSI	0.788 (0.708–0.867)	31.129	0.656	0.833	0.489
HOMA-IR	0.766 (0.680–0.852)	2.750	0.877	0.583	0.460
FPG (mg/dL)	0.662 (0.558–0.766)	90.500	0.960	0.306	0.265

CI, confidence interval.

**Figure 4:**
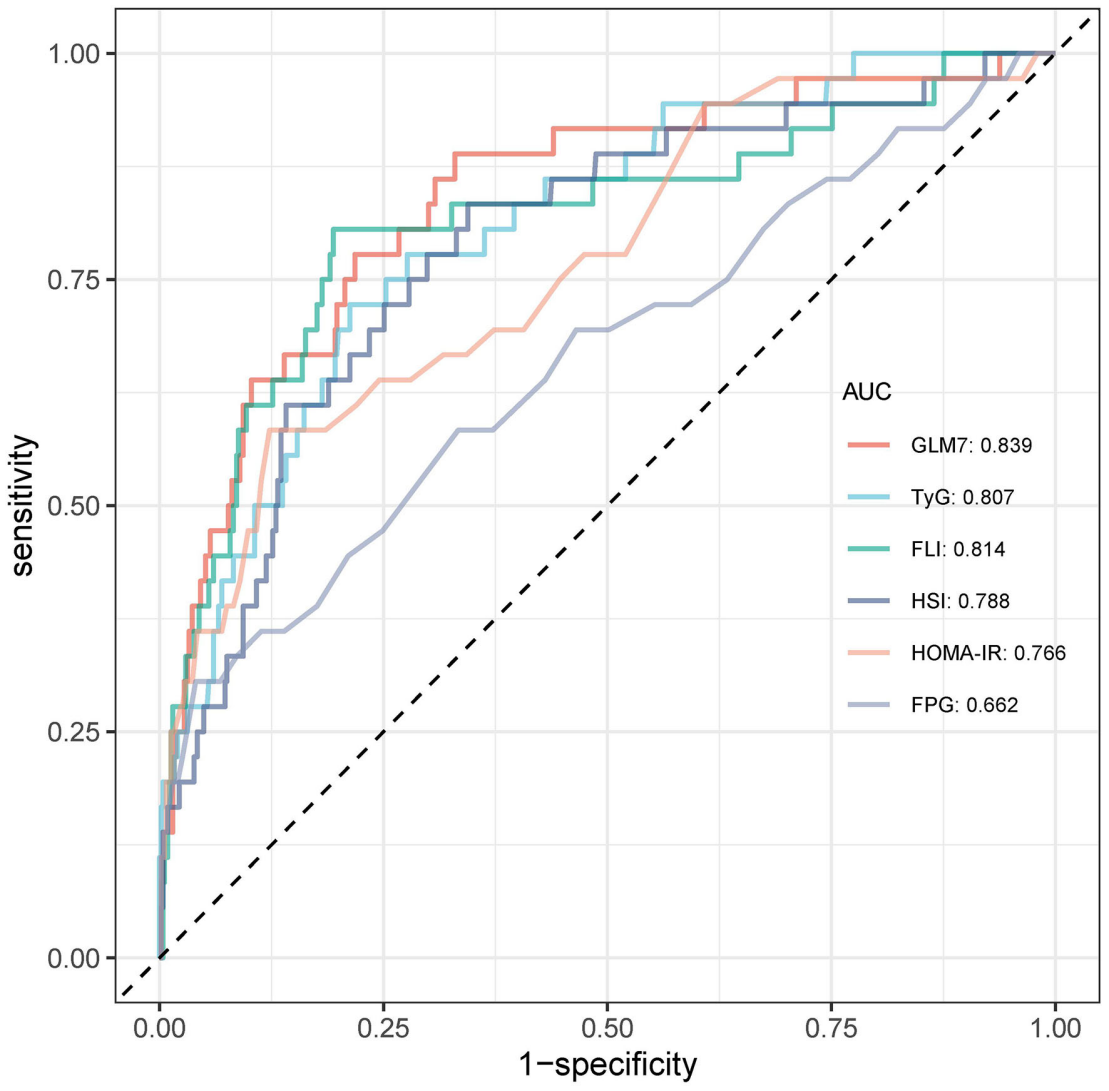
ROC curves comparing the discriminatory performance of GLM7 with TyG, FLI, HSI, HOMA-IR, and FPG for subsequent GDM. GLM7 showed the highest observed AUC of 0.839 among the evaluated metabolic markers.

Because GLM7 incorporates FPG as one of its constituent variables, these adjusted analyses should be regarded as exploratory and should not be interpreted as demonstrating discriminatory performance independent of the FPG component.

## 4 Discussion

In this prospective cohort study, higher first-trimester GLM7 levels were independently associated with subsequent GDM, with a fully adjusted OR of 14.27 (95% CI 5.01–40.67). The association exhibited a positive linear dose-response relationship and remained generally consistent across the subgroup and sensitivity analyses. GLM7 also demonstrated favorable discriminative performance within this cohort, with an AUC of 0.839. Collectively, these findings support further evaluation of the previously developed GLM7 index as a candidate composite metabolic marker for early GDM risk stratification. However, they should be interpreted as an initial pregnancy-specific evaluation of association and discrimination rather than as evidence supporting a validated clinical prediction tool.

Previous perinatal studies have reported inconsistent associations and variable discriminative performance for individual lipid markers, hepatic indices, and IR markers in relation to GDM (47–51). In the present cohort, women who subsequently developed GDM had higher levels of FPG, fasting insulin, and HOMA-IR, supporting the relevance of early disturbances in glucose metabolism and IR (52). Nevertheless, individual biomarkers may incompletely capture the complex metabolic alterations preceding GDM, which involve IR, adiposity, dysregulated lipid metabolism, and impaired glucose homeostasis (3, 6, 53, 54). These observations provide a rationale for evaluating composite indices that integrate multiple metabolic domains (12, 55, 56). Although GLM7 achieved the highest observed AUC, DeLong's test demonstrated statistically significant differences only compared with HOMA-IR and FPG, whereas no significant differences were observed compared with the TyG index, FLI, or HSI. Therefore, the present findings should not be interpreted as demonstrating the universal superiority of GLM7 over established composite metabolic indices (57). The slightly higher Youden index observed for FLI does not contradict the higher AUC of GLM7, because the Youden index reflects diagnostic performance at a single operating threshold, whereas the AUC summarizes discriminative performance across all possible thresholds.

The observed association is biologically plausible because GLM7 integrates anthropometric, glycemic, insulinemic, and lipid-related variables that reflect multiple metabolic pathways implicated in GDM development. During pregnancy, metabolic adaptations in the liver and adipose tissue contribute to changes in insulin sensitivity, hepatic glucose production, lipid mobilization, and circulating FFA concentrations (24, 58, 59). Excess hepatic fat accumulation and impaired insulin signaling may promote lipid overflow, systemic IR, and subsequent glucose intolerance. These metabolic disturbances may be further amplified by inflammatory and adipokine-mediated pathways (10, 11, 60–65). By combining age, pre-pregnancy BMI, FPG, fasting insulin, TG, LDL, and HDL, GLM7 may capture a broader spectrum of early metabolic alterations than any individual laboratory marker (66). Composite metabolic indices have been extensively investigated in relation to NAFLD, T2DM, and cardiometabolic multimorbidity in non-pregnant populations, but their application in early pregnancy remains relatively underexplored (17–20, 25). The present study therefore provides an initial independent evaluation of the previously developed GLM7 index in a pregnancy-specific population, with subsequent GDM as the study outcome.

The analyses treating GLM7 as a continuous variable, together with the trend and RCS analyses, consistently supported a positive linear association between GLM7 and subsequent GDM. Although the fully adjusted tertile analysis did not show a strictly monotonic increase across all exposure categories, the effect estimate for the intermediate tertile was imprecise, as reflected by its wide CI. Given that only 36 GDM events occurred, categorization of GLM7 further reduced the number of events within each exposure category, thereby increasing random variability and reducing statistical power. The apparent non-monotonic pattern observed across tertiles is more likely to reflect limited statistical power and random variation than a true departure from the overall association. Similarly, the subgroup analyses produced wider CIs because of the limited number of events within individual strata. These analyses were intended primarily to assess the consistency of the association rather than to provide precise stratum-specific estimates. No statistically significant interactions were observed for age, HOMA-IR, pre-pregnancy BMI, or HS, providing no evidence of meaningful effect modification.

The ROC findings should be interpreted with caution. The primary ROC analyses assessed the discriminative performance of individual biomarkers and composite indices rather than that of fully developed multivariable prediction models. Direct comparisons of GLM7 with individual biomarkers are inherently influenced by the greater amount of metabolic information incorporated into the seven-component GLM7 formula. Comparisons with the TyG index, FLI, HSI, and HOMA-IR are likewise influenced by differences in the number and types of variables included in each index. Thus, the higher observed AUC of GLM7 may partly reflect its broader metabolic composition rather than intrinsic superiority. Covariate-adjusted ROC analyses retained a broadly similar numerical ranking of the evaluated markers; however, none of the pairwise differences remained statistically significant after adjustment. This finding further supports a cautious interpretation of the comparative discriminative performance of GLM7. Moreover, this study was not designed to determine the incremental discriminative performance or clinical utility of GLM7. Larger independent studies are needed to determine whether GLM7 provides additional discrimination beyond established maternal characteristics, first-trimester FPG, and existing composite metabolic indices, and whether any improvement is accompanied by better calibration or greater clinical utility (67, 68).

An important methodological consideration is that FPG is one of the components of GLM7 and is independently associated with subsequent GDM. This does not constitute strict outcome circularity because GLM7 was calculated using measurements obtained at 10–14 weeks of gestation, whereas GDM was diagnosed later at 24–28 weeks using a standardized 75-g OGTT according to the IADPSG criteria. GLM7 and the GDM outcome were therefore assessed at different stages of pregnancy using distinct measurement procedures. Nevertheless, biological overlap remains unavoidable, and the inclusion of first-trimester FPG likely contributed to both the observed association and the discriminative performance of GLM7. To place this contribution into context, FPG alone yielded an AUC of 0.662 in this cohort, which was substantially lower than that of the complete GLM7 index (AUC 0.839), suggesting that the non-FPG components contribute additional metabolic information.

However, the estimated association (fully adjusted OR 14.27) likely reflects, at least in part, the contribution of the FPG component, and the relatively high AUC should not be interpreted as demonstrating discrimination fully independent of FPG. A recent methodological commentary by Wang and Huang (69) also questioned the inclusion of fasting glucose within GLM7 when evaluating diabetes-related outcomes because of potential overlap between the predictor and outcome. Although the present study examined future OGTT-defined GDM rather than concurrent diabetes, this concern remains relevant. Future studies should quantify the incremental contribution of GLM7 beyond first-trimester FPG and other established metabolic indices.

This study has several strengths. The prospective cohort design established a clear temporal sequence between first-trimester GLM7 assessment and subsequent GDM diagnosis. Covariate selection was informed by DAGs and prior evidence, while the number of adjusted covariates was restricted in view of the limited number of outcome events. Multicollinearity was minimal, with all VIF values below 1.2. Multiple sensitivity analyses, prespecified subgroup analyses, RCS modeling, and E-value analyses were also used to evaluate the robustness of the findings.

Several limitations should be acknowledged. First, the observational design precludes causal inference. Second, only 36 participants developed GDM, resulting in wide CIs and limited statistical precision, particularly in the tertile and subgroup analyses. Although the number of adjusted covariates was constrained according to the events-per-variable principle, residual overfitting and model instability cannot be excluded. Third, this study evaluated associations and discriminative performance but neither developed nor externally validated a clinical prediction model. Model calibration, internal validation, clinical net benefit, incremental predictive value, and decision-analytic performance were not assessed. Moreover, the GLM7 threshold of 6.932 was both derived and evaluated within the same cohort and may therefore represent an optimistic estimate. Accordingly, it should be regarded as exploratory rather than as an established clinical decision threshold. Fourth, the complete-case analysis excluded 41 of 623 otherwise eligible participants. Although the proportion of excluded participants was relatively small, comparison of the included and excluded participants showed that most baseline characteristics were similar between the two groups; however, excluded participants had modestly higher first-trimester FPG and FFA levels. Therefore, some degree of selection bias cannot be excluded if missingness was correlated with metabolic status or GDM risk. Because missing GDM outcomes could not be reliably imputed, multiple imputation was not performed. Fifth, the cohort consisted exclusively of Korean women with singleton pregnancies, which may limit the generalizability of the findings to other ethnic populations and obstetric settings. Finally, GLM7 was calculated from single measurements of FPG and fasting insulin obtained during early pregnancy. Both biological and analytical variability may influence classification around the proposed threshold, and a single assessment cannot capture longitudinal metabolic changes throughout gestation. Therefore, external validation in larger, multicentre, multi-ethnic cohorts with repeated measurements is required before GLM7 or its proposed threshold can be considered for routine screening or individualized risk assessment.

## 5 Conclusion

In conclusion, this study provides an initial pregnancy-specific evaluation of the previously developed GLM7 index. Higher first-trimester GLM7 levels were independently associated with subsequent GDM and demonstrated favorable discrimination within this Korean cohort. These findings support further evaluation of GLM7 as a candidate composite marker for early GDM risk stratification. However, because this study did not develop or validate a clinical prediction model, external validation, assessment of calibration, incremental discriminatory value, clinical utility, and confirmation of the proposed GLM7 cut-off in independent populations are required before clinical implementation.

## Data Availability

The data analyzed in this study are derived from an earlier study by Lee et al and served as the primary source for this research: https://doi.org/10.1371/journal.pone.0221400.

## References

[1] Modzelewski R Stefanowicz-Rutkowska MM Matuszewski W Bandurska-Stankiewicz EM. Gestational diabetes mellitus-recent literature review. J Clin Med. (2022) 11:5736. doi: 10.3390/jcm11195736 36233604 PMC9572242

[2] Page KA Luo S Wang X Chow T Alves J Buchanan TA . Children exposed to maternal obesity or gestational diabetes mellitus during early fetal development have hypothalamic alterations that predict future weight gain. Diabetes Care. (2019) 42:1473–80. doi: 10.2337/dc18-2581 31332028 PMC6647040

[3] Sweeting A Wong J Murphy HR Ross GP. A clinical update on gestational diabetes mellitus. Endocr Rev. (2022) 43:763–93. doi: 10.1210/endrev/bnac003 35041752 PMC9512153

[4] Johns EC Denison FC Norman JE Reynolds RM. Gestational diabetes mellitus: mechanisms, treatment, and complications. Trends Endocrinol Metab. (2018) 29:743–54. doi: 10.1016/j.tem.2018.09.004 30297319

[5] Metzger BE Gabbe SG Persson B Buchanan TA Catalano PA Damm P . International association of diabetes and pregnancy study groups recommendations on the diagnosis and classification of hyperglycemia in pregnancy. Diabetes Care. (2010) 33:676–82. doi: 10.2337/dc10-0719 20190296 PMC2827530

[6] McIntyre HD Catalano P Zhang C Desoye G Mathiesen ER Damm P . Gestational diabetes mellitus. Nat Rev Dis Primers. (2019) 5:47. doi: 10.1038/s41572-019-0098-8 31296866

[7] Gregory GA Robinson TIG Linklater SE Wang F Colagiuri S de Beaufort C . Global incidence, prevalence, and mortality of type 1 diabetes in 2021 with projection to 2040: a modeling study. Lancet Diabetes Endocrinol. (2022) 10:741–60. doi: 10.1016/S2213-8587(22)00218-2 36113507

[8] Mitanchez D Burguet A Simeoni U. Infants born to mothers with gestational diabetes mellitus: mild neonatal effects, a long-term threat to global health. J Pediatr. (2014) 164:445–50. doi: 10.1016/j.jpeds.2013.10.076 24331686

[9] Lowe WL Jr Scholtens DM Kuang A Linder B Lawrence JM Lebenthal Y . Hyperglycemia and adverse pregnancy outcome follow-up study (HAPO FUS): maternal gestational diabetes mellitus and childhood glucose metabolism. Diabetes Care. (2019) 42:372–80. doi: 10.2337/dc18-1646 30655380 PMC6385693

[10] Hillier TA Pedula KL Ogasawara KK Vesco KK Oshiro CES Lubarsky SL . A pragmatic, randomized clinical trial of gestational diabetes screening. N Engl J Med. (2021) 384:895–904. doi: 10.1056/NEJMoa2026028 33704936 PMC9041326

[11] Haque MM Tannous WK Herman WH Immanuel J Hague WM Teede H . Cost-effectiveness of diagnosis and treatment of early gestational diabetes mellitus: economic evaluation of the TOBOGM study, an international multicenter randomized controlled trial. EClinicalMedicine. (2024) 71:102610. doi: 10.1016/j.eclinm.2024.102610 38813447 PMC11133791

[12] Yang MN Zhang L Wang WJ Huang R He H Zheng T . Prediction of gestational diabetes mellitus by multiple biomarkers at early gestation. BMC Pregnancy Childbirth. (2024) 24:601. doi: 10.1186/s12884-024-06651-4 39285345 PMC11406857

[13] Savvidou M Nelson SM Makgoba M Messow CM Sattar N Nicolaides K. First-trimester prediction of gestational diabetes mellitus: examining the potential of combining maternal characteristics and laboratory measures. Diabetes. (2010) 59:3017–22. doi: 10.2337/db10-0688 20876721 PMC2992761

[14] Miettinen HE Rönö K Koivusalo S Stach-Lempinen B Pöyhönen-Alho M Eriksson JG . Elevated serum squalene and cholesterol synthesis markers in pregnant obese women with gestational diabetes mellitus. J Lipid Res. (2014) 55:2644–54. doi: 10.1194/jlr.P049510 25301963 PMC4242456

[15] Jin C Lin L Han N Zhao Z Liu Z Luo S . Effects of dynamic change in fetuin-A levels from the first to the second trimester on insulin resistance and gestational diabetes mellitus: a nested case-control study. BMJ Open Diabetes Res Care. (2020) 8:e000802. doi: 10.1136/bmjdrc-2019-000802 31958310 PMC7039607

[16] Yao X Chen X Adam REH Zhang Z Ge Y Li Y . Higher serum adrenomedullin concentration is associated with an increased risk of gestational diabetes mellitus: a nested case-control study in Wuhan, China. Nutr Res. (2022) 107:117–27. doi: 10.1016/j.nutres.2022.09.004 36215885

[17] Wang J Xu C Xun Y Lu Z Shi J Yu C . ZJU index: a novel model for predicting nonalcoholic fatty liver disease in a Chinese population. Sci Rep. (2015) 5:16494. doi: 10.1038/srep16494 26568423 PMC4645098

[18] Zheng K Yin Y Guo H Ma L Liu R Zhao T . Association between the ZJU index and risk of new-onset non-alcoholic fatty liver disease in non-obese participants: a Chinese longitudinal prospective cohort study. Front Endocrinol. (2024) 15:1340644. doi: 10.3389/fendo.2024.1340644 38405152 PMC10884868

[19] Wu C Loh YH Huang H Xu C. ZJU index as a predictive tool for diabetes incidence: insights from a population-based cohort study. Diabetes Metab Syndr Obes. (2024) 17:715–24. doi: 10.2147/DMSO.S446042 38371391 PMC10873143

[20] Zhang H Li X Lian X Sun J Wang X Zhang M . The ZJU index as a predictor of all-cause mortality in metabolic syndrome patients: a prospective cohort study from NHANES 2005-2018. Medicine. (2025) 104:e44203. doi: 10.1097/MD.0000000000044203 40898499 PMC12401420

[21] Lee SM Cho GJ Wi WY Norwitz ER Koo BK Lee J . Metabolic dysfunction-associated fatty liver disease as a risk factor for adverse outcomes in subsequent pregnancy: a nationwide cohort study. Hepatol Int. (2023) 17:367–76. doi: 10.1007/s12072-022-10458-w 36542262

[22] Zhao B Han X Meng Q Luo Q. Early second trimester maternal serum markers in the prediction of gestational diabetes mellitus. J Diabetes Investig. (2018) 9:967–74. doi: 10.1111/jdi.12798 29288571 PMC6031488

[23] Koralegedara IS Warnasekara JN Dayaratne KG De Silva FN Premadasa JK Agampodi SB. Non-alcoholic fatty liver disease (NAFLD): a significant predictor of gestational diabetes mellitus (GDM) and early pregnancy miscarriages-prospective study in Rajarata pregnancy cohort (RaPCo). BMJ Open Gastroenterol. (2022) 9:e000831. doi: 10.1136/bmjgast-2021-000831 35193889 PMC8867246

[24] Eslam M Newsome PN Sarin SK Anstee QM Targher G Romero-Gomez M . A new definition for metabolic dysfunction-associated fatty liver disease: an international expert consensus statement. J Hepatol. (2020) 73:202–9. doi: 10.1016/j.jhep.2020.07.045 32278004

[25] Wang Z Chen S Feng X Chen X Evans PC Strijdom H . GLM7—A novel composite glycolipid index derived from routine health indicators for enhanced diagnosis and prediction of multimorbidity. Adv Sci. (2025) 12:e10552. doi: 10.1002/advs.202510552 40874812 PMC12622479

[26] Lee SM Kim BJ Koo JN Norwitz ER Oh IH Kim SM . Nonalcoholic fatty liver disease is a risk factor for large-for-gestational-age birthweight. PLoS ONE. (2019) 14:e0221400. doi: 10.1371/journal.pone.0221400 31449538 PMC6709883

[27] von Elm E Altman DG Egger M Pocock SJ Gøtzsche PC Vandenbroucke JP. The strengthening the reporting of observational studies in epidemiology (STROBE) statement: guidelines for reporting observational studies. Int J Surg. (2014) 12:1495–9. doi: 10.1016/j.ijsu.2014.07.013 25046131

[28] Saadeh S Younossi ZM Remer EM Gramlich T Ong JP Hurley M . The utility of radiological imaging in nonalcoholic fatty liver disease. Gastroenterology. (2002) 123:745–50. doi: 10.1053/gast.2002.35354 12198701

[29] Wallace TM Levy JC Matthews DR. Use and abuse of HOMA modeling. Diabetes Care. (2004) 27:1487–95. doi: 10.2337/diacare.27.6.1487 15161807

[30] Zhang C Catalano P. Screening for gestational diabetes. JAMA. (2021) 326:487–9. doi: 10.1001/jama.2021.12190 34374733

[31] Heinemann L. Insulin assay standardization: leading to measures of insulin sensitivity and secretion for practical clinical care: response to Staten et al. Diabetes Care. (2010) 33:e83. doi: 10.2337/dc10-0034 20508228

[32] Simental-Mendía LE Rodriguez-Morán M Guerrero-Romero F. The product of fasting glucose and triglycerides as surrogate for identifying insulin resistance in apparently healthy subjects. Metab Syndr Relat Disord. (2008) 6:299–304. doi: 10.1089/met.2008.0034 19067533

[33] Gayoso-Diz P Otero-González A Rodriguez-Alvarez MX Gude F García F De Francisco A . Insulin resistance (HOMA-IR) cut-off values and the metabolic syndrome in a general adult population: effect of gender and age: EPIRCE cross-sectional study. BMC Endocr Disord. (2013) 13:47. doi: 10.1186/1472-6823-13-47 24131857 PMC4016563

[34] Bedogni G Bellentani S Miglioli L Masutti F Passalacqua M Castiglione A . The fatty liver index: a simple and accurate predictor of hepatic steatosis in the general population. BMC Gastroenterol. (2006) 6:33. doi: 10.1186/1471-230X-6-33 17081293 PMC1636651

[35] Lee JH Kim D Kim HJ Lee CH Yang JI Kim W . Hepatic steatosis index: a simple screening tool reflecting nonalcoholic fatty liver disease. Dig Liver Dis. (2010) 42:503–8. doi: 10.1016/j.dld.2009.08.002 19766548

[36] Gao Y Hu Y Xiang L. Remnant cholesterol, but not other cholesterol parameters, is associated with gestational diabetes mellitus in pregnant women: a prospective cohort study. J Transl Med. (2023) 21:531. doi: 10.1186/s12967-023-04322-0 37544989 PMC10405385

[37] Zhang J Suo Y Wang L Liu D Jia Y Fu Y . Association between atherogenic index of plasma and gestational diabetes mellitus: a prospective cohort study based on the Korean population. Cardiovasc Diabetol. (2024) 23:237. doi: 10.1186/s12933-024-02341-9 38970008 PMC11227226

[38] Feeney T Hartwig FP Davies NM. How to use directed acyclic graphs: guide for clinical researchers. BMJ. (2025) 388:e078226. doi: 10.1136/bmj-2023-078226 40118502

[39] An Z Niu T Lu Y Yao B Feng F Zhang H . Nonlinear association between alanine aminotransferase to high-density lipoprotein cholesterol ratio and risk of gestational diabetes mellitus. Sci Rep. (2024) 14:24872. doi: 10.1038/s41598-024-76656-8 39438670 PMC11496691

[40] Song S Son DH Baik SJ Cho WJ Lee YJ. Triglyceride glucose-waist circumference (TyG-WC) is a reliable marker to predict non-alcoholic fatty liver disease. Biomedicines. (2022) 10:2251. doi: 10.3390/biomedicines10092251 36140352 PMC9496145

[41] Lai J Liu Z Wang H Kong X Wei Y Cao Y . Assessment of seven insulin resistance surrogate indexes for predicting cardiometabolic multimorbidity among Chinese middle-aged and older adults: a national prospective cohort study. Cardiovasc Diabetol. (2026) 25:168. doi: 10.1186/s12933-026-03168-2 41937148 PMC13067570

[42] Ma S Jiang M Xuan Y Shen Y Chen K Li B . Associations of eight insulin resistance-related indices and genetic risk with incident cardiometabolic multimorbidity among participants with hypertension: a large prospective cohort study. Cardiovasc Diabetol. (2026) 25:154. doi: 10.1186/s12933-026-03154-8 41913237 PMC13159231

[43] Altman DG. Practical Statistics for Medical Research. London: Chapman & Hall (1991). doi: 10.1201/9780429258589

[44] Santos Ferreira DL Williams DM Kangas AJ Soininen P Ala-Korpela M Smith GD . Association of pre-pregnancy body mass index with offspring metabolic profile: analyses of 3 European prospective birth cohorts. PLoS Med. (2017) 14:e1002376. doi: 10.1371/journal.pmed.1002376 28829768 PMC5568725

[45] Lisonkova S Muraca GM Potts J Liauw J Chan WS Skoll A . Association between prepregnancy body mass index and severe maternal morbidity. JAMA. (2017) 318:1777–86. doi: 10.1001/jama.2017.16191 29136442 PMC5820710

[46] Haneuse S VanderWeele TJ Arterburn D. Using the E-value to assess the potential effect of unmeasured confounding in observational studies. JAMA. (2019) 321:602–3. doi: 10.1001/jama.2018.21554 30676631

[47] Pratama S Aulia RD Jazilah I Lauren BC. Liver enzyme biomarkers before or in early pregnancy as predictors for gestational diabetes mellitus: a systematic review and meta-analysis. J ASEAN Fed Endocr Soc. (2026) 41:170–85. doi: 10.15605/jafes.041.01.5807 42311541 PMC13269346

[48] Abdalla MMI Ismail-Khan M. Liver as a metabolic sensor in gestational diabetes: implications for offspring's liver and diabetes risk. World J Hepatol. (2025) 17:110185. doi: 10.4254/wjh.v17.i11.110185 41368113 PMC12683350

[49] Tumurbaatar B Poole AT Olson G Makhlouf M Sallam HS Thukuntla S . Adipose tissue insulin resistance in gestational diabetes. Metab Syndr Relat Disord. (2017) 15:86–92. doi: 10.1089/met.2016.0124 28080219 PMC5333579

[50] Trivett C Lees ZJ Freeman DJ. Adipose tissue function in healthy pregnancy, gestational diabetes mellitus and pre-eclampsia. Eur J Clin Nutr. (2021) 75:1745–56. doi: 10.1038/s41430-021-00948-9 34131300 PMC8636251

[51] Vrachnis N Belitsos P Sifakis S Dafopoulos K Siristatidis C Pappa KI . Role of adipokines and other inflammatory mediators in gestational diabetes mellitus and previous gestational diabetes mellitus. Int J Endocrinol. (2012) 2012:549748. doi: 10.1155/2012/549748 22550485 PMC3328961

[52] Borges Manna L Syngelaki A Würtz P Koivu A Sairanen M Pölönen T . First-trimester nuclear magnetic resonance-based metabolomic profiling increases the prediction of gestational diabetes mellitus. Am J Obstet Gynecol. (2025) 233:71.e1–14. doi: 10.1016/j.ajog.2024.12.019 39694165

[53] Yang D Shen Y Shuai R. Association between AST/ALT ratio and the risk of gestational diabetes mellitus in Korean pregnant women. PLoS ONE. (2025) 20:e0331484. doi: 10.1371/journal.pone.0331484 40880520 PMC12396687

[54] You Y Hu H Cao C Han Y Tang J Zhao W. Association between the triglyceride to high-density lipoprotein cholesterol ratio and the risk of gestational diabetes mellitus: a second analysis based on data from a prospective cohort study. Front Endocrinol. (2023) 14:1153072. doi: 10.3389/fendo.2023.1153072 37576966 PMC10415043

[55] Wei J Ma N Bai L Lu Q. Fatty liver index in early pregnancy predicts the risk of gestational diabetes mellitus. Front Endocrinol. (2026) 17:1659379. doi: 10.3389/fendo.2026.1659379 41837136 PMC12979087

[56] Rathnayake H Han L da Silva Costa F Paganoti C Dyer B Kundur A . Advancement in predictive biomarkers for gestational diabetes mellitus diagnosis and related outcomes: a scoping review. BMJ Open. (2024) 14:e089937. doi: 10.1136/bmjopen-2024-089937 39675825 PMC11647389

[57] Takele WW Vesco KK Josefson J Redman LM Hannah W Bonham MP . Effective interventions in preventing gestational diabetes mellitus: a systematic review and meta-analysis. Commun Med. (2024) 4:75. 10.1038/s43856-024-00491-1 PMC11032369 38643248

[58] Bae JC. Metabolic dysfunction-associated steatotic liver disease: the role of hepatic steatosis in insulin resistance and metabolic health. Endocrinol Metab. (2025) 40:304–6. doi: 10.3803/EnM.2025.2340 40123045 PMC12061749

[59] Lee SM Kwak SH Koo JN Oh IH Kwon JE Kim BJ . Non-alcoholic fatty liver disease in the first trimester and subsequent development of gestational diabetes mellitus. Diabetologia. (2019) 62:238–48. doi: 10.1007/s00125-018-4779-8 30470912

[60] Du H Li D Molive LM Wu N. Advances in free fatty acid profiles in gestational diabetes mellitus. J Transl Med. (2024) 22:180. doi: 10.1186/s12967-024-04922-4 38374136 PMC10875910

[61] Sivan E Boden G. Free fatty acids, insulin resistance, and pregnancy. Curr Diab Rep. (2003) 3:319–22. doi: 10.1007/s11892-003-0024-y 12866995

[62] Liang X Lai K Li X Ren D Gui S Li Y . Association between triglyceride glucose-body mass index and gestational diabetes mellitus: a prospective cohort study. BMC Pregnancy Childbirth. (2025) 25:170. doi: 10.1186/s12884-025-07294-9 39962434 PMC11834603

[63] Leca BM Lagojda L Kite C Karteris E Kassi E Randeva HS . Maternal obesity and metabolic (dysfunction) associated fatty liver disease in pregnancy: a comprehensive narrative review. Expert Rev Endocrinol Metab. (2024) 19:335–48. doi: 10.1080/17446651.2024.2365791 38860684

[64] Hamedi-Shahraki S Klisic A Amirkhizi F Mercantepe F. Association of the triglyceride-glucose index with inflammatory markers and dysregulation of adipokines in patients with metabolic syndrome. BMC Endocr Disord. (2026) 26:44. doi: 10.1186/s12902-025-02142-5 41546009 PMC12892619

[65] Xin Z Feng L Yu Q Shi Y Tang T Tong X . Triglyceride glucose waist circumference and non alcoholic fatty liver disease: a systematic review and meta analysis. BMC Gastroenterol. (2025) 25:328. doi: 10.1186/s12876-025-03888-x 40312323 PMC12044922

[66] Duo Y Song S Qiao X Zhang Y Xu J Zhang J . A simplified screening model to predict the risk of gestational diabetes mellitus in pregnant Chinese women. Diabetes Ther. (2023) 14:2143–57. doi: 10.1007/s13300-023-01480-8 37843770 PMC10597926

[67] Meertens LJE van Montfort P Scheepers HCJ . External validation and clinical utility of prognostic prediction models for gestational diabetes mellitus: a prospective cohort study. Acta Obstet Gynecol Scand. (2020) 99:665–74. doi: 10.1111/aogs.13811 31955406 PMC7317858

[68] Van Calster B Vickers AJ. Calibration of risk prediction models: impact on decision-analytic performance. Med Decis Making. (2015) 35:162–9. doi: 10.1177/0272989X14547233 25155798

[69] Wang Y Huang TY. Comment on: “GLM7—A novel composite glycolipid index derived from routine health indicators for enhanced diagnosis and prediction of multimorbidity”. Adv Sci. (2026) 13:e74610. doi: 10.1002/advs.74610 41770882 PMC13073296

