# Supplementary material for: Association of the GLM7 composite metabolic index with gestational diabetes mellitus in early pregnancy: a secondary analysis of a Korean prospective pregnancy cohort
Source: Front Nutr. 2026 Sep 15;13:1907985. doi: 10.3389/fnut.2026.1907985 (PMC13619892; doi:10.3389/fnut.2026.1907985)
Supplement: Supplementary file 1 [file Data_Sheet_1.docx]

**Supplementary Figure S1** Directed acyclic graphs. FPG, fasting plasma glucose; ALT, alanine aminotransferase; AST, aspartate aminotransferase; TG, triglyceride; GGT, gamma-glutamyltransferase; TC, total cholesterol; HDL, high-density lipoprotein cholesterol; LDL, low-density lipoprotein cholesterol; Pre-pregnancy BMI, pre-pregnancy body mass index; HOMA-IR, homeostasis model assessment-insulin resistance; FFA, free fatty acids; GDM, gestational diabetes mellitus.

**Supplementary Figure S2** Covariate-adjusted ROC curves for GLM7 and comparator metabolic markers in relation to subsequent GDM. Each metabolic marker (GLM7, TyG, FLI, HSI, HOMA-IR, or FPG) was added individually to an identical base model, including parity, TC, FFA, and ADPN. The AUC is shown for each adjusted model.

**Supplementary Table S1** Baseline characteristics of included and excluded participants.

| **Characteristics** | **Total** | **Included** | **Excluded** | ***p* value** |
| --- | --- | --- | --- | --- |
| Participants | 623 | 582 | 41 |  |
| Age (years) | 32.1 ± 3.9 | 32.1 ± 3.8 | 32.0 ± 5.0 | 0.906 |
| BMI (kg/m^2^) | 22.1 ± 3.5 | 22.0 ± 3.5 | 22.6 ± 3.7 | 0.317 |
| AST (IU/L) | 17.8 ± 8.1 | 17.8 ± 8.1 | 18.5 ± 6.4 | 0.709 |
| ALT (IU/L) | 11.0 (8.0, 15.0) | 11.0 (8.0, 15.0) | 13.0 (7.5, 16.5) | 0.765 |
| GGT (IU/L) | 14.0 ± 8.6 | 14.0 ± 8.5 | 14.7 ± 10.9 | 0.714 |
| TC (mg/dL) | 173.1 ± 27.3 | 172.9 ± 27.1 | 177.9 ± 32.6 | 0.414 |
| TG (mg/dL) | 119.5 ± 48.2 | 119.0 ± 47.5 | 131.4 ± 65.1 | 0.249 |
| HDL (mg/dL) | 65.0 ± 13.6 | 64.9 ± 13.5 | 66.3 ± 14.9 | 0.638 |
| LDL (mg/dL) | 84.1 ± 21.9 | 84.1 ± 21.7 | 85.3 ± 27.9 | 0.805 |
| FPG (mg/dL) | 77.1 ± 9.7 | 76.9 ± 9.7 | 82.4 ± 8.4 | 0.013 |
| Insulin (μIU/mL) | 8.4 (5.4, 11.6) | 8.4 (5.4, 11.6) | 9.7 (7.1, 13.2) | 0.178 |
| FFA (μEq/L) | 654.1 ± 276.5 | 648.3 ± 272.3 | 823.3 ± 346.8 | 0.005 |
| parity |  |  |  | 0.453 |
| No | 324 (52.0) | 305 (52.4) | 19 (46.3) |  |
| Yes | 299 (48.0) | 277 (47.6) | 22 (53.7) |  |
| NAFLD |  |  |  | 0.923 |
| No | 505 (81.1) | 472 (81.1) | 33 (80.5) |  |
| Yes | 118 (18.9) | 110 (18.9) | 8 (19.5) |  |
| ADPN (ng/mL) | 5016.1 (2811.6, 7941.6) | 5026.2 (2821.3, 8051.0) | 4363.9 (2739.2, 6366.2) | 0.323 |
| HOMA-IR | 1.5 (1.0, 2.3) | 1.5 (1.0, 2.3) | 2.0 (1.5, 2.4) | 0.117 |
| Hepatic steatosis |  |  |  | 0.416 |
| Grade 0 | 505 (81.1) | 472 (81.1) | 33 (80.5) |  |
| Grade 1 | 90 (14.4) | 85 (14.6) | 5 (12.2) |  |
| Grade 2 | 20 ( 3.2) | 17 (2.9) | 3 (7.3) |  |
| Grade 3 | 8 ( 1.3) | 8 (1.4) | 0 (0) |  |
| FLI | 11.5 (6.1, 22.8) | 11.5 (6.1, 22.7) | 14.7 (8.9, 40.5) | 0.277 |
| HSI | 30.4 ± 4.8 | 30.3 ± 4.7 | 30.7 ± 5.3 | 0.747 |
| GDM |  |  |  | 0.413 |
| No | 571 (93.6) | 546 (93.8) | 25 (89.3) |  |
| Yes | 39 ( 6.4) | 36 (6.2) | 3 (10.7) |  |

BMI, body mass index; TG, triglyceride; TC, total cholesterol; HDL, high-density lipoprotein cholesterol; LDL, low-density lipoprotein cholesterol; FPG, fasting plasma glucose; ALT, alanine aminotransferase; GGT, gamma-glutamyl transferase; AST, aspartate aminotransferase; FFA, free fatty acid; NAFLD, nonalcoholic fatty liver disease; ADPN, adiponectin; HOMA-IR, homeostatic model assessment of insulin resistance; FLI, fatty liver index; HSI, hepatic steatosis index; GDM, gestational diabetes mellitus. Values are reported as mean±SD, median (Q1-Q3), or N (%). NAFLD was defined as hepatic steatosis grade ≥ 1 on ultrasonography.

**Supplementary Table S2** Variance Inflation Factor.

| **Variables** | **VIF** |
| --- | --- |
| GLM7 | 1.152 |
| FFA | 1.021 |
| TC | 1.076 |
| ADPN | 1.059 |
| parity | 1.036 |

**Supplementary Table S3 Box-Tidwell Test for Assessment of Linear Relationship Between GLM7 and GDM Risk.**

| **Term** | **Coefficient** | ***p*_value** |
| --- | --- | --- |
| GLM7*ln(GLM7) | 10.133 | 0.326 |

**Supplementary Table S4** Subgroup analysis of the association between **GLM7** and GDM risk.

| **Subgroup** | **Total** | **Event (%)** | **adj.OR**  **(95% CI)** | **adj.*p* value** | ***p* for interaction** |
| --- | --- | --- | --- | --- | --- |
| **Age(years)** |  |  |  |  | 0.422 |
| <35 | 446 | 29 (6.5) | 16.96 (4.93~58.36) | <0.001 |  |
| ≥35 | 136 | 7 (5.1) | 14.38 (1.43~144.14) | 0.023 |  |
| **BMI**  **(kg/m^2^)** |  |  |  |  | 0.684 |
| <25 | 486 | 17 (3.5) | 7.05 (1.72~28.9) | 0.007 |  |
| ≥25 | 96 | 19 (19.8) | 15.72 (2.35~104.92) | 0.004 |  |
| **HOMA-IR** |  |  |  |  | 0.109 |
| ≤2 | 385 | 12 (3.1) | 3.02 (0.4~22.61) | 0.283 |  |
| >2 | 197 | 24 (12.2) | 200.55 (21.36~1882.78) | <0.001 |  |
| **Hepatic steatosis** |  |  |  |  | 0.332 |
| Grade 0 | 472 | 16 (3.4) | 7.31 (1.86~28.72) | 0.004 |  |
| Grade 1-3 | 110 | 20 (18.2) | 17.11 (2.09~139.91) | 0.008 |  |

**Supplementary Table S5** Pairwise DeLong comparisons of unadjusted and

covariate-adjusted ROC curves.

| **(A) Unadjusted ROC** | | |
| --- | --- | --- |
| **Comparison** | **ΔAUC** | **DeLong *p*** |
| GLM7 vs TyG | 0.032 | 0.314 |
| GLM7 vs FLI | 0.025 | 0.413 |
| GLM7 vs HSI | 0.051 | 0.151 |
| GLM7 vs HOMA-IR | 0.073 | 0.002 |
| GLM7 vs FPG | 0.177 | 0.002 |
| **(B) Covariate-adjusted ROC** | | |
| **Comparison** | **ΔAUC** | **DeLong *p*** |
| GLM7 vs TyG | 0.008 | 0.709 |
| GLM7 vs FLI | 0.018 | 0.455 |
| GLM7 vs HSI | 0.024 | 0.344 |
| GLM7 vs HOMA-IR | 0.033 | 0.175 |
| GLM7 vs FPG | 0.043 | 0.104 |
